# Bodyweight and Weightlifting Exercise Injury Burden: National Analysis from 2014 to 2023

**DOI:** 10.1055/a-2866-5672

**Published:** 2026-06-11

**Authors:** Jason Liu, Mina Ghali, Matthew Aceto, Vraj Amin, Samuel Hovland, Franz Jones

**Affiliations:** 1 Orthopedics 124506 University of Central Florida College of MedicineOrlandoFloridaUnited States; 2 Physical Medicine and Rehabilitation 2814 Nova Southeastern UniversityFort LauderdaleFloridaUnited States

**Keywords:** exercise, weightlifting, injury

## Abstract

Bodyweight exercises and weightlifting are foundational to strength training, yet their relative public health burdens remain poorly defined in large, nationally representative samples. We analyzed data from the National Electronic Injury Surveillance System from 2014 to 2023. A standardized keyword screening protocol was utilized to identify cases of weightlifting and bodyweight injuries treated in US emergency departments. Variance estimation was performed using Taylor series linearization to account for the complex, stratified sampling design and generate nationally representative estimates. Over the 10-year study period, national estimates of 546,655 weightlifting-related injuries and 108,001 bodyweight-related injuries were identified. Weightlifting injuries primarily involved the trunk (70.1%) and phalanges (13.7%). Bodyweight injuries were significantly associated with upper and lower extremity trauma (20.7%) and joint dislocations (8.7%). After adjusting for age and sex in a cluster-robust multivariable model, exercise modality was not a significant predictor of inpatient hospitalization (adjusted odds ratio: 0.91; 95% confidence interval: 0.60–1.38; *p* =0.666). While weightlifting poses a higher absolute volume burden, there is no significant difference in hospitalization odds when adjusting for age and sex. Prevention should focus on a suitable technique for heavy lifts and safe joint positioning for dynamic bodyweight movements, with increased diagnostic precaution and safety monitoring recommended for older participants.

## Introduction

Physical activity is a cornerstone of health, with exercise modalities such as bodyweight exercises and weightlifting gaining significant popularity for their efficacy in improving musculoskeletal health, metabolic function, and bone mineral density. 1​ 2​ 3​ ​4​ However, as participation in these activities increases, there has been a corresponding increase in exercise-related injuries presenting to acute care settings. 1​ ​5​ While bodyweight exercises are often perceived as lower-risks due to minimal equipment requirements, 6​ weightlifting involves technical complexity and external loads that may increase the mechanical stress on the skeletal system. Incorporation of weightlifting into individual fitness regimens has demonstrated increased muscle mass and bone mineral density, increased insulin sensitivity, and decreased blood pressure, among numerous other health benefits. 7​ Despite these benefits, there are risks associated with weightlifting. The added load and technical complexity of weightlifting are thought to be the cause of higher injury frequency. 8​ Despite these perceptions, comparative studies on injury patterns between these two exercise modalities remain limited, particularly in large, nationally representative samples.

Many previous studies have evaluated musculoskeletal injuries in weightlifting, yet comparative studies utilizing large, nationally representative samples remain scarce. The studies on “bodyweight exercise” injuries frequently utilize vague terminology, often conflating resistance-based movements with cardiovascular activities or flexibility training. 9​ ​10​ This study aims to address these gaps by utilizing a standardized, keyword-based screening protocol of the National Electronic Injury Surveillance System (NEISS) to compare the national injury burden, demographic distributions, and hospitalization patterns between weightlifting and bodyweight training from 2014 to 2023. By identifying modality-specific injury patterns, these findings may assist clinicians and fitness professionals in developing safer, evidence-based exercise guidelines tailored to specific training environments.

## Methods

This cross-sectional, descriptive epidemiologic study analyzed exercise-related injuries using data from the U.S. Consumer Product Safety Commission’s National Electronic Injury Surveillance System (NEISS) between January 1, 2014 and December 31, 2023. The NEISS is a stratified probability sample of approximately 100 US emergency departments (EDs) and provides nationally representative estimates of injuries associated with consumer products and recreational activities. National estimates and corresponding 95% confidence intervals (CIs) were calculated using NEISS-provided statistical weights to account for the complex survey design.

Injuries were identified using product codes for weightlifting equipment (3,265) and exercises without equipment (3,299). A standardized narrative screening protocol was applied to the case narratives (Appendix Table 1 ):

**Table:** **Table 1** Weighted demographic characteristics of injuries (2014–2023)

Variables	Weightlifting	Bodyweight
Weighted national estimate	546,655	108,001
Age (y)		
Mean (SD)	30.0 (23.1)	30.1 (16.5)
Median	25	27
Gender		
Male	76.4%	62.3%
Female	23.6%	37.7%

Abbreviations: NEISS, National Electronic Injury Surveillance System; SD, standard deviation.

Note: National estimates utilize NEISS-provided statistical weights.

**Weightlifting group:** Defined as resistance training utilizing external loads (e.g., free weights or machines) to improve musculoskeletal strength. Inclusion required narratives to specify an active lifting mechanism (e.g., “bench press” and “deadlift”) rather than passive contact, like “tripped over weights” which were excluded.
**Bodyweight group:** Defined as resistance movements where the participant’s own body mass serves as the primary resistive load. This category includes calisthenics and plyometric strength movements (e.g., “push-ups” and “burpees”) but excludes activities focused on aerobic conditioning (e.g., running), flexibility (e.g., yoga), or low-impact postural control (e.g., Pilates).

All analyses incorporated NEISS-provided sampling weights to generate nationally representative estimates. Variance estimation was performed using Taylor series linearization. This method accounts for the complex, stratified survey design by utilizing the NEISS-provided stratum and primary sampling unit variables. To identify independent predictors of inpatient hospitalization, a cluster-robust multivariable logistic regression model was employed, adjusting for exercise modality, age, and sex simultaneously.

To evaluate longitudinal patterns and address year-to-year variations, temporal trend testing was performed using a weighted Poisson regression model. This analysis calculated the average annual percentage change (AAPC) and its 95% confidence interval for the total study period (2014–2023) and a specific subset representing the COVID-19 pandemic period (2018–2021). This approach allowed for a formal statistical assessment of whether observed fluctuations in injury burden represented significant temporal trends rather than sampling variations.

All analyses were conducted using weighted data to reflect the national injury burden, but unweighted sample counts are presented for transparency. Statistical significance was defined as a two-sided *p* -value of<0.05. All analyses were performed using R (version 4.0.1).

## Results

Between 2014 and 2023, the screening protocol identified 13,563 weightlifting cases and 2,837 bodyweight cases with a national estimate of 546,655 weightlifting-related injuries and 108,001 bodyweight exercise–related injuries treated in US EDs. Across the study period, weightlifting accounted for a consistently higher ED-treated injury burden compared with bodyweight exercise. The mean age of injured individuals was similar between modalities (weightlifting: 30.0 y; bodyweight: 30.1 y). Men accounted for a greater proportion of injuries in both groups, representing 76.4% of weightlifting injuries and 62.3% of bodyweight exercise injuries ( Table 1 ).

**Fig. 1:**
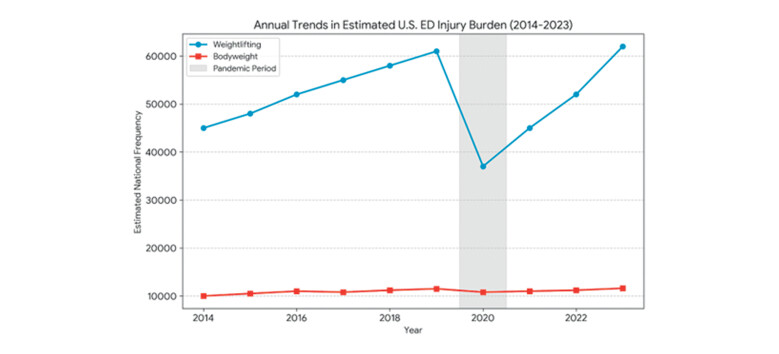
Annual trends in the estimated US emergency department injury burden (2014–2023). This line graph illustrates the annual national injury burden for weightlifting (blue) and bodyweight exercise (red) over a 10-year period. Data points represent weighted national estimates derived from the National Electronic Injury Surveillance System (NEISS).

A marked decline in weightlifting-related injuries was observed during the COVID-19 pandemic, with a 39.4% reduction in 2020, followed by a rebound that exceeded pre-pandemic levels by 2023. In contrast, bodyweight exercise injuries remained relatively stable over time, with a 6.0% decline during the pandemic period ( Fig. 1 ). Longitudinal analysis using weighted Poisson regression demonstrated a non-significant overall trend in weightlifting-related injuries over the 10-year study period (AAPC: 0.8%; 95% CI:−1.2% to 2.9%; and *p* =0.412). However, a distinct, localized decrease was observed between 2019 and 2020, coinciding with the onset of the COVID-19 pandemic, followed by a return to baseline burden by 2022. Upon further review, a subset trend analysis of the COVID-19 period (2018–2021) using weighted Poisson regression demonstrated a significant AAPC of−4.7% (95% CI:−7.2% to−2.1% and *p* =0.014). This confirms that the decline in injury burden during 2020 was a statistically significant deviation from the baseline 10-year trend. Longitudinal analysis of bodyweight-related injuries from 2014 to 2023 demonstrated a stable trend (AAPC:−1.1%; 95% CI:−3.4% to 1.3%; and *p* =0.354). Similar to the weightlifting cohort, a significant temporal decline was observed within the bodyweight group during the 2018–2021 pandemic subset analysis (AAPC:−6.2%; 95% CI:−9.8% to−2.4%; and *p* =0.008).

**Fig. 2:**
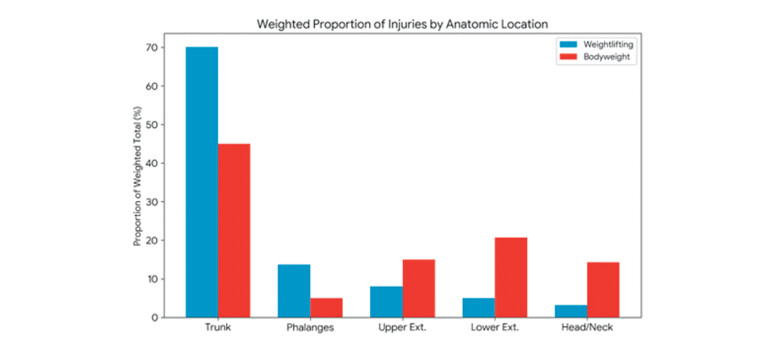
Weighted proportion of injuries by anatomic location. A grouped bar chart comparing the relative distribution of injuries by body region for weightlifting and bodyweight exercises. Weightlifting injuries were disproportionately concentrated in the trunk (70.1%) and phalanges (13.7%). Bodyweight exercise injuries demonstrated a significantly higher relative involvement of the lower extremities (20.7%) compared to the weightlifting cohort.

Distinct injury patterns were observed between exercise modalities. Weightlifting injuries more frequently involved the trunk (70.1%) and phalanges (13.7%), whereas bodyweight exercise injuries were more commonly associated with lower extremity involvement (20.7%) and joint dislocations (8.7%; **Figs.** 2 **and** 3 ).

**Fig. 3:**
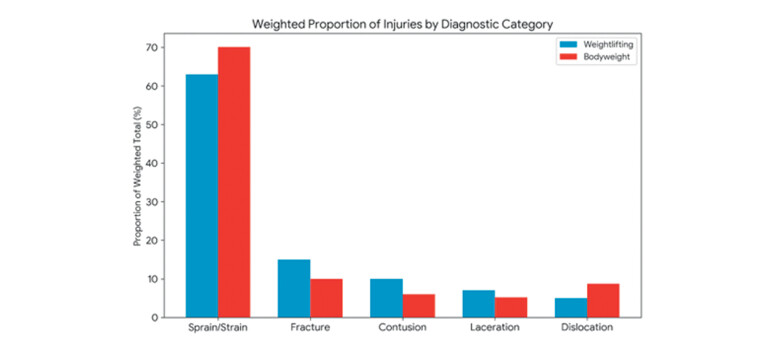
Weighted proportion of injuries by diagnostic category. Distribution of primary injury diagnoses presenting to US emergency departments for both exercise modalities. While sprains and strains represent the leading diagnosis for both groups, the bodyweight exercise was associated with a higher relative proportion of joint dislocations (8.7%).

Although the absolute burden of weightlifting-related injuries was greater, multivariable weighted logistic regression demonstrated that hospitalization risk was primarily associated with increasing age rather than exercise modality. After adjustment for age and sex, weightlifting was not independently associated with higher odds of inpatient hospitalization compared with the bodyweight exercise (aOR 0.91; 95% CI: 0.60–1.38; and *p* =0.666). Each additional year of age was associated with increased odds of hospitalization (aOR 1.03; 95% CI, 1.023–1.044; and *p* <0.001), while sex was not a significant predictor ( Table 2 ).

**Table:** **Table 2** Adjusted odds ratios (aOR) for inpatient hospitalization

Variables	aOR	95% Confidence interval	*p* -Value
Modality (weightlifting)	0.91	(0.60–1.38)	0.666
Age (per year increase)	1.03	(1.02–1.04)	<0.001
Sex (male)	1.17	(0.78–1.74)	0.442

Note: Model adjusted for modality, age, and sex simultaneously. Primary sampling unit-clustered standard errors account for hospital-level variations. Reference groups: bodyweight exercise (modality) and female (sex).

## Discussion

This study provides a comprehensive analysis of the injury burden associated with weightlifting and bodyweight exercises in the United States from 2014 to 2023. Our findings highlight that while weightlifting accounts for a significantly higher absolute volume of ED visits, the clinical severity measured by the odds of inpatient hospitalization is statistically comparable between the two modalities once age and sex are accounted for in multivariable modeling.

Weightlifting-related injuries consistently outnumbered bodyweight injuries across the 10-year study period. Interestingly, weighted Poisson regression confirmed that the COVID-19 pandemic resulted in a statistically significant decline in the injury burden for both weightlifting (AAPC:−4.7%, *p* =0.014) and bodyweight (AAPC:−6.2%, *p* =0.008) exercises between 2018 and 2021. This suggests that the public health impact of the pandemic was not limited to equipment-based training but extended to calisthenics, likely due to the widespread closure of community fitness centers and public calisthenic parks.

Men accounted for the majority of injuries in both groups, a disparity potentially linked to higher participation rates in strength training and a higher likelihood of engaging in high-intensity competition. 11​ ​12​ Furthermore, physiological differences, such as greater muscle mass and absolute strength in males, may facilitate the use of heavier loads, which increases the risk of soft tissue trauma. 11​

While sprains and strains were the primary diagnosis for both modalities, weightlifting disproportionately involved the trunk and phalanges. Trunk injuries in weightlifters often coincide with improper lifting techniques or overexertion or exercises such as squats and deadlifts. 13​ These injury patterns may reflect biomechanical demands, where certain resistance exercises generate substantial compressive and shear forces on the lumbar spine. 14​ Trunk injuries in weightlifters likely reflect the significant biomechanical demands of compound movements such as squats and deadlifts. These exercises generate substantial compressive and shear forces on the lumbar spine, involving trunk flexion and rotation. 14​ Previous research on heavy deadlifts demonstrates that spinal loads are highly variable to lever arms and personal anatomy; an improper technique or overexertion during these high-load activities can exacerbate the risk of acute soft tissue trauma or chronic low back pain. 13​

Phalangeal injuries likely stem from equipment-related trauma, such as dropped weights or crush injuries from heavy loads. 1​ Acute trauma of the phalanges, such as fractures, and overuse injuries, like tendinitis, are prevalent among weightlifters due to repetitive gripping and manipulation of weight and may exacerbate phalangeal injuries. 15​

In contrast, bodyweight exercises were associated with a higher proportion of upper and lower extremity injuries and joint dislocations. These patterns may be attributed to the dynamic, high-impact nature of movements like plyometrics, which can place abnormal stress on the shoulders, wrists, and knees if performed with an improper technique. 16​ ​17​ Excessive force or abnormal joint positioning through the improper technique during these movements can increase the risk of joint dislocations particularly in the shoulders, wrists, and knees. 18​

Our cluster-robust multivariable model indicated that exercise modality was not an independent predictor of inpatient hospitalization (aOR: 0.91; 95% CI: 0.60–1.38; and *p* =0.666), suggesting that while the absolute volume of weightlifting injuries is higher, the odds of severe outcomes do not differ significantly between these modalities when adjusting for age and sex. However, it is important to acknowledge that hospitalization odds are not a perfect proxy for injury severity. Admission decisions are likely influenced by a complex interplay of non-clinical factors, including institutional admission thresholds, patient insurance status, and access to emergency care, which cannot be fully captured through surveillance narratives. The finding that older individuals had significantly higher odds of hospitalization (aOR: 1.03; 95% CI: 1.02–1.04; and *p* <0.001) requires nuanced interpretation. While physiological factors such as decreased bone mineral density and slower soft-tissue healing contribute to injury complexity in older adults, these data likely also reflect heightened diagnostic precautions among emergency clinicians, leading to older individuals more likely to be admitted and worked up in the inpatient setting. 1​ ​19​ Consequently, the significant increased burden in older populations should be taken in context with clinical prudence and access to care.

There are several limitations to this study that necessitate a cautious interpretation of the findings. As a system-level surveillance tool, the NEISS database captures only injuries treated in EDs, thereby excluding injuries managed in primary care, sports medicine clinics, or through self-care. Furthermore, it may under-represent injuries sustained in elite or professional sport settings where specialized team clinicians manage care on-site. Crucially, this study reports on the injury burden rather than the comparative risk. The participation denominators, such as the total number of individuals performing each modality or the frequency and intensity of training, are not available in the NEISS database. Therefore, we cannot conclude that one modality is inherently “safer” than the other. Furthermore, the data lack granular context regarding the training load, participant experience level, or whether the exercises were performed under professional supervision. The narratives also do not provide explicit details on the exercise form or the specific biomechanical mechanism of injury. Consequently, the anatomical patterns discussed, such as the trunk dominance in weightlifting, should be viewed as observed associations within the national burden rather than direct evidence of causal biomechanical failure. While our standardized keyword-based protocol was designed to be rigorous, the potential for coding misclassification at the institutional level remains an inherent limitation of surveillance data. Our keyword-based approach may represent a conservative underestimation of the bodyweight injury burden, as vague narratives were excluded to maintain specificity. Hospitalization was used as an outcome measure in this study; however, it should not be interpreted as a direct surrogate for injury severity alone. Admission decisions may also be influenced by patient comorbidities, diagnostic uncertainty, access to inpatient care, institutional admission thresholds, and social factors not captured within the NEISS dataset.

## Conclusions

Between 2014 and 2023, weightlifting-related injuries accounted for a significantly higher absolute national burden compared to bodyweight exercises in the United States. While weightlifting injuries were more frequent, the exercise modality was not an independent predictor of inpatient hospitalization after adjusting for age and sex. Instead, the age emerged as the primary driver of severe outcomes, likely reflecting a combination of physiological vulnerability, accidental mechanisms, and heightened diagnostic precaution in older populations. Clinicians and fitness professionals should recognize the distinct injury patterns associated with each modality, such as trunk and equipment-related trauma in weightlifting versus joint dislocations and extremity injuries in bodyweight training to better tailor safety recommendations and guide safe participation across the lifespan.

## Supplementary Material

Supplementary Material Zusatzmaterial
