# Supplementary material for: Bodyweight and Weightlifting Exercise Injury Burden: National Analysis from 2014 to 2023
Source: Sports Med Int Open. 2026 Jun 11;10:a28665672. doi: 10.1055/a-2866-5672 (PMC13295158; doi:10.1055/a-2866-5672)
Supplement: Supplementary file 1 — Supplementary Material [file 10-1055-a-2866-5672_28844808.pdf]

Category	Inclusion Keywords (Narrative)	Exclusion Keywords (Narrative)
Weightlifting	weight, dumbbell, barbell, bench press, deadlift, kettlebell, lifting	Equipment failure, bystanders, tripped over weights
Bodyweight	pushup, push-up, pullup, pull-up, plank, burpee, situp, sit-up, calisthenics	Running, jogging, yoga, Pilates, stretching, walking, hiking

Appendix Table 1. Narrative Screening Keyword Protocol for Exercise Modalities. Inclusion and exclusion criteria utilized for standardized case identification from NEISS narratives.

Sports Medicine International Open | DOI 10.1055/a-2866-5672 | © The Author(s).
